# Reply to Ayoub-Charette et al. Lack of Biological Plausibility and Major Methodological Issues Cast Doubt on the Association between Aspartame and Autism. Comment on “Fowler et al. Daily Early-Life Exposures to Diet Soda and Aspartame Are Associated with Autism in Males: A Case-Control Study. *Nutrients* 2023, *15*, 3772”

**DOI:** 10.3390/nu16050676

**Published:** 2024-02-28

**Authors:** Sharon Parten Fowler, David Gimeno Ruiz de Porras, Michael D. Swartz, Paula Stigler Granados, Lynne Parsons Heilbrun, Raymond F. Palmer

**Affiliations:** 1 Department of Medicine, Joe R. & Teresa Lozano Long School of Medicine, The University of Texas Health Science Center at San Antonio, San Antonio, TX 78229, USA; 2 Center for Research in Occupational Health (CiSAL), Universitat Pompeu Fabra, 08003 Barcelona, Spain; gimeno@uthscsa.edu; 3 Center for Biomedical Research in Epidemiology and Public Health Network (CIBER) of Epidemiology and Public Health (CIBERESP), 28029 Madrid, Spain; 4 Department of Environmental and Occupational Health, UT School of Public Health San Antonio, The University of Texas Health Science Center at San Antonio, San Antonio, TX 78229, USA; 5 Department of Biostatistics and Data Science, School of Public Health, The University of Texas Health Science Center at Houston, Houston, TX 77030, USA; michael.d.swartz@uth.tmc.edu; 6 Division of Environmental Health, San Diego State University School of Public Health, San Diego, CA 92182, USA; pstiglergranados@sdsu.edu; 7 Department of Epidemiology, Human Genetics, and Environmental Sciences, School of Public Health in San Antonio, The University of Texas Health Science Center at Houston, San Antonio, TX 78229, USA; 8 Department of Family Practice and Community Medicine, Joe R. & Teresa Lozano Long School of Medicine, The University of Texas Health Science Center at San Antonio, San Antonio, TX 78229, USA; palmerr@uthscsa.edu

Thank you for the opportunity to respond to the concerns raised by Ayoub-Charette et al. [1]. As they disclosed, the senior authors of this Comment have longstanding relationships with industry associations that promote the manufacture and sale of non-nutritive sweeteners (NNS), diet sodas, and other diet products. Four of the five authors have disclosed conflicts of interest directly related to the promotion and/or manufacture and sale of NNS and products containing them. The intermingling of industrial and academic priorities in this group is concerning; we therefore appreciate the opportunity to respond to their comments.

Overarching issues:

Before addressing specific concerns raised by the authors of the Comment, we would repeat and emphasize a key statement from the conclusions section of our paper [2]: These associations do not prove causality. Our data were collected through a case–control study, the limitations of which were previously described in our paper and are reiterated below in response to specific comments by Ayoub-Charette et al. Given these limitations, our results—which are consistent with earlier prospective cohort studies that found increased health risks among offspring gestationally exposed to these substances—raise a warning flag about maternal consumption of these products during pregnancy and breastfeeding but cannot prove causality.

Nonetheless, taking a historical perspective, it should be noted that case–control studies, in general, are often used as a first step in the epidemiologic search for the causes of rare diseases. Case–control studies, for example, provided early warnings of the link between cigarette smoking and lung cancer before more definitive data on these associations became available through prospective cohort studies. (Please see White’s 1990 discussion of the relative roles of case–control and prospective cohort studies in identifying cigarette smoking as a major causative agent of lung cancer [3]). As White described, in studying the association between cigarette smoking and lung cancer, the epidemiologic study designs that were employed progressed from small case–control studies to larger, meticulously designed case–control studies and then on to large-scale prospective cohort studies [3]. Using this historical sequence by way of context, our study would reside within the category of small case–control studies, with the limitations we already described. In the earlier investigations into cigarette smoking and lung cancer, small case–control studies identified the association between smoking and lung cancer as early as 1939, but it was not until two larger case–control studies replicating these findings were published in 1950 that the associations were taken more seriously. As White described, associations between cigarette smoking and lung cancer—identified through case–control studies—were next studied through prospective cohort studies, the results of which were critical in moving medical and public health opinion to accept “…the disturbing finding that a pleasant and popular habit was, in the long term, potentially lethal. Conviction that this was so did not come easily” [3]. Thus, these investigators followed a classic progression: from studying associations using case–control studies to studying them within prospective cohort studies, results from which were less influenced by several limitations of case–control studies.

Beyond prospective cohort studies, the ideal, gold-standard epidemiologic study design for the evaluation of the relationship between an exposure and an outcome is a clinical intervention, the classic example of which is a randomized controlled trial (RCT). In the classic RCT, the exposure is hypothesized to be a beneficial one, which can prevent or reduce the health burden from the condition under study. There are two main arguments against the use of an RCT in evaluating the associations reported from our study, however: the first is ethical, and the second is logistical. In the first category, based on results from both animal studies and case–control and cohort studies in humans, it is our assessment that it would be unethical to conduct clinical interventions in which soon-to-be pregnant women were randomized to a study arm in which they were instructed to consume diet beverages, NNS in general, or aspartame specifically, daily, in dosages comparable to those found in our study. Indeed, based upon results from earlier studies in children, Sylvetsky et al. recently wrote, “We propose that the FDA restrict NSSs [non-sugar-sweeteners] in products marketed to children until definitive evidence of benefit and lack of harm is available” [4]. We agree with the concerns expressed by these investigators and feel that these concerns are equally valid regarding exposing an embryo or fetus in utero to these products and their metabolites. As to the second issue—logistical difficulties—it would be enormously challenging and expensive to conduct an RCT to study these associations, and also, by our calculations, it might take up to seven years or more to conclude such a study and finish data analyses. Thus, for both reasons, conducting an RCT to study this question would be problematic.

This, therefore, raises the question of which next steps could be pursued to provide more definitive answers to these questions more rapidly. Because data are already available from large-scale prospective cohort studies, we would recommend analyses of these data, with adjustment for additional potential confounders, to further explore these associations without the limitations of our own study. Meta-analyses of results from multiple cohort studies could be used to further evaluate these associations. Meanwhile, analyses of existing data from individual prospective cohort studies would be an important next step in evaluating the associations that emerged from our study.

Response to specific concerns raised by Ayoub-Charette et al.:

With regard to the limitations in our study data noted by Ayoub-Charette et al., it is worth restating two that we had previously described in our paper. First, we could not include maternal body mass index, diabetes status, and other maternal cardiometabolic risk factors as covariates in our analyses and thus cannot exclude the possibility that our results may have been confounded by reverse causality: maternal overweight/obesity and diabetes themselves increase the risk of autism and could increase a woman’s motivation to use diet products. In our sensitivity analyses, the daily consumption of non-nutritive sweeteners was no higher within key sociodemographic strata previously associated with an increased risk of overweight/obesity and diabetes, but adjustments for individual cardiometabolic risk factors will be critical for future analyses of prospectively collected data. Second, all data were recorded retrospectively, years after the diagnosis of autism or autism spectrum disorder (ASD). Future analyses of prospectively collected dietary data are warranted to evaluate these associations without these limitations.

We thank Ayoub-Charette et al. for the opportunity to affirm that aspartame is indeed rapidly metabolized within the small intestine into aspartic acid (40%), phenylalanine (50%), and methanol (10%) [5], which, in turn, is converted into its highly reactive first-phase metabolite, formaldehyde, a known neurotoxin [6]. Formaldehyde can be further metabolized into formic acid. For this reason, unlike other NNS detected in human amniotic fluid [7], cord blood [7], and breast milk [8], aspartame itself is not detectable in these fluids. Instead, within and beyond the small intestine, it is the aspartame metabolites and the molecules, bacteria, and tissues with which they interact that would affect the cecal and gut microbiota. Along with aspartame metabolites themselves, other metabolites produced by the gut microbiota [9,10,11] also enter the bloodstream and, thence, the tissues, organs, and fluids beyond.

Ayoub-Charette et al. expressed concerns about the lack of plausible mechanisms at biologically relevant exposure levels underlying our findings. From multiple studies in both animals and humans, however, plausible biological mechanisms for our findings emerged from two overarching patterns in aspartame-fed animals and their offspring: adverse impacts on intracellular metabolic processes and adverse impacts on the gut microbiota. Because of the questions raised by Ayoub-Charette et al., these two categories of mechanisms are described below. Unless otherwise noted, references were provided in our paper [2].

## 1. Disruption of One-Carbon Metabolism (OCM); Lowered Availability of Reduced Glutathione (Glutamine Sulfhydryl: GSH); Decreased Antioxidant, Detoxification, and Methylation Capacity; and Increased Oxidative Stress in Aspartame-Fed Animals

A pre-eminent impact of aspartame consumption in animals was a decreased availability of GSH, a major antioxidant critical for a wide range of functions within neurons and throughout the body [2]. GSH protects cells from oxidative stress, provides detoxification protection from both endogenous and exogenous toxins, and supports methylation processes [2]. GSH availability is maintained by one-carbon metabolism (OCM), three interrelated metabolic pathways that support the supply of multiple molecules necessary for defense against toxins and oxidative stress, methylation, and other critical cellular processes [12]. Animals fed aspartame at dosages within normal human consumption ranges [13] demonstrated the following: strikingly decreased levels of GSH, with significantly reduced antioxidant and detoxification capacities [2,13,14,15,16]; significantly increased oxidative stress, including elevated levels of lipid peroxidation and mitochondrial dysfunction [2]; and disruptions of processes within OCM [15], which could explain the GSH depletion and each of the findings above.

These findings are strongly congruent with metabolic patterns observed among individuals with autism [12]: decreased GSH availability and total antioxidant capacity; reduced detoxification capacity; and increased oxidative stress, including increased mitochondrial dysfunction [17,18,19,20]. Autism typically emerges through the overlaying of toxic or other adverse exposures upon individual genetic susceptibility – or, as Heilbrun et al. wrote, “‘dose plus host’ determines toxicity” [21]. Through its deleterious impacts on OCM—thereby simultaneously reducing antioxidant and detoxification capacities while increasing toxicant load—aspartame could contribute to the perfect storm which underlies the etiology of autism. Importantly, it could leave exposed individuals more vulnerable to environmental toxins known to increase the risk of autism.

Ayoub-Charette et al. stated, however, that, at normal consumption dosages, the metabolites of aspartame would not enter the maternal gut or bloodstream in sufficient concentrations to cause harm to gestationally exposed offspring. To examine this question, it is useful to consider the results from several early studies in both animals and humans. These evaluated the impact of aspartame consumption on circulating levels of two of its metabolites—methanol and phenylalanine—using a dose of 34 mg of aspartame/kg body weight (mg/kg), a dosage representing the upper limit (99th percentile) of normal consumption in the general population. For comparison, U.S. and European acceptable daily intake (ADI) levels are 40 and 50 mg/kg, respectively. When this dosage was fed in a single serving to one-year-old infants [22] and to adults in two studies [23,24], their plasma phenylalanine concentrations increased within 30 to 45 min by 90% [22], 54% [23], and 96% [24], respectively, and remained significantly elevated until 2 h after the dose [24].

Increases in serum methanol concentrations were even more dramatic. In rats [25] and adult humans [23] fed the same dosage, serum methanol concentrations increased by over 130% [25] and by 170% [23], respectively, within 30 to 60 min and remained significantly elevated for three hours [25]. In another study, adult males were fed a lower dose of aspartame: 500 mg total, the FDA’s estimated average daily intake of aspartame in humans—equivalent to about 2.8 cans of diet cola—which represented a dosage of approximately 6 to 8.7 mg/kg in the male volunteers in this study [25]. Following this lower dose of aspartame, serum methanol concentrations rose more than 50% above basal levels within 45 min and remained significantly elevated three hours following the dosage [25]. These dosages, given in a single serving, would result in higher individual spikes of circulating phenylalanine and methanol than the average consumer would experience following each of multiple servings throughout the day. Nonetheless, these results suggest a greater impact of aspartame consumption on circulating levels of methanol than the 1% to 10% range reported by Ayoub-Charette et al. In addition, the magnitude of each temporary elevation of circulating metabolites might provide a better indicator of their potential metabolic impacts, compared with the mean overall percentage of their contribution, averaged over 24 h. (Please note variations in phenylalanine concentrations reported by Stegink et al. in 1988, for example [26]).

A different approach to evaluating the impact of aspartame consumption, however, is to examine its actual impact on tissues. Our paper already described the adverse metabolic impacts of aspartame consumption in a number of studies [2]. In addition, however, a 2023 study [27] published after our paper reported that pregnant mice treated with aspartame by oral gavage at doses as low as 3.5 mg/kg (comparable to 1.4 cans of diet cola consumed by a 160 lb woman) experienced impaired placental growth and function, with significantly reduced placental and fetal weight [27]. The investigators also demonstrated a significantly increased production of reactive oxygen species, downregulation of expression of the antioxidant MnSOD, increased intracellular oxidative stress, and diminished cellular proliferation in placental trophoblasts exposed to phenylalanine in vitro in concentrations comparable to 10 mg/kg of aspartame [27]. Thus, using biologically relevant doses, this recent study further demonstrated adverse metabolic impacts from aspartame consumption or exposure – in this case, on the placenta itself [2,13].

As Choudhary and Pretorius wrote, “The existing animal studies and the limited human studies suggest that aspartame and its metabolites, whether consumed in quantities significantly higher than the recommended safe dosage or within recommended safe levels, may disrupt the oxidant/antioxidant balance, induce oxidative stress, and damage cell membrane integrity, potentially affecting a variety of cells and tissues and causing a deregulation of cellular function, ultimately leading to systemic inflammation” [13].

## 2. Adverse Impacts on the Gut Microbiota

As many as 90% of children with ASD have been estimated to experience gastrointestinal disorders [28], which may exacerbate other symptoms of autism [29]. Animal studies demonstrated the following impacts of aspartame on the gut microbiota, including several which were found in individuals with autism [30]: increases in total bacteria and the abundance of *Enterobacteriaceae* and *Clostridium leptum* [10], significantly decreased lactose fermentation and increased abundance of *Clostridium cluster IV* [9], and reduced diversity within the microbiota [31]. Disturbances in biodiversity within the microbiome have been reported to cause increased permeability of the gut [28], which can increase inflammation systemically, including within the central nervous system [28]. Aspartame and two other leading NNSs—sucralose and saccharin— have also increased oxidative stress and apoptosis in intestinal epithelial cells and increased epithelial barrier permeability [32], which would further exacerbate inflammation systemically. Inflammation itself has been reported to increase autism risk [33].

Investigators have also observed alterations in the production of gut metabolites, including increased production of the cecal short-chain fatty acids (SCFA) propionate, butyrate, and isobutyrate in aspartame-fed Sprague–Dawley dams, as well as changes in the cecal microbiome of their offspring, resulting in an increased abundance of propionate-producing strains in the offspring gut microbiome [34]. Aspartame-fed rat dams exhibited a doubling of serum concentrations of propionate [10], which the investigators hypothesized resulted from increased production of propionate by colon microbiota [10]. Propionate and other SCFAs have been hypothesized to trigger ASD [35]. As we noted earlier [2], elevated propionate concentrations have been associated with increased permeability of both the gut and the blood–brain barrier [35,36]. This combination would allow increased access of toxins from the gut into the bloodstream and, thence, into the brain, as well as throughout the body. Not surprisingly, therefore, elevated serum propionate levels have been associated with decreased GSH levels and increased oxidative stress, excitotoxicity, and neuroinflammation [28,37,38] and with an increased risk of autism in several studies [10,35,36,37,38,39].

Aspartame-fed animals have also exhibited significantly increased serum concentrations of kynurenine [11], another metabolite produced by the gut microbiota which is elevated in individuals with autism [28,33,35,40]. Elevated kynurenine has been hypothesized to cause increased excitotoxicity and neuroinflammation [33] and to increase the risk of neuroanatomical problems characteristic of ASD, which arise during fetal brain development [41].

Animal studies have demonstrated that maternal aspartame consumption can adversely impact not only the gut microbiota but also weight gain and glucose tolerance in both the dams themselves and their gestationally exposed offspring who did not themselves consume aspartame [9]. Germ-free mice that received fecal microbiota transplants (FMT) from these aspartame-exposed offspring subsequently developed the same cardiometabolic problems the offspring had experienced [9]. That these adverse impacts can be replicated through interspecies FMT from offspring offers strong evidence that maternal aspartame consumption provokes deleterious changes in the offspring’s microbiota.

Aspartame consumption has also had adverse impacts on gut microbiota in humans and their offspring. Among healthy volunteers fed aspartame for two weeks, significant microbiome composition and functional changes, and heightened glycemic responses were noted in some volunteers—termed ‘responders’—and changes were observed in their plasma metabolome [11]. Germ-free mice that received FMTs from these ‘responders’ developed similarly elevated glycemic responses [11]. In another study, three-month-old human infants whose mothers consumed diet beverages daily during pregnancy exhibited increased concentrations of spermidine and succinate—urine metabolites produced in the gut by the metabolism of putrescine—compared with the offspring of mothers who rarely or never consumed these beverages [42]. Urinary succinate concentrations were associated with increased body mass index in this study [42]. These studies in both animals and humans demonstrate adverse changes in the gut microbiota resulting from consumption of, or gestational exposure to, aspartame and other NNS.

Some patterns found in the gut microbiota and metabolites in aspartame- and NNS-exposed animals are similar to those observed in individuals with ASD. As noted earlier, an increased abundance of *Clostridium* species, for example [43,44], and a lower percentage of *Akkermansia* [29] have been found in individuals with ASD, as well as among aspartame- and NNS-exposed animals. Because our primary, dichotomized exposure variable was daily early-life exposure to ≥1 diet soda/day, and aspartame, Ace K, and sucralose are the three leading NNS used in diet sodas [45], it should be noted that sucralose [11,46] and Ace K [47] have both also been found in multiple studies to have adverse impacts on the gut microbiota [11,46,47,48]. One study found that the consumption of low doses of sucralose and Ace K by pregnant and lactating mice resulted in significant changes in the gut microbiota and associated processes in their pups [49]. These included significant increases in *Firmicutes* and decreases in *Akkermansia muciniphila* in the gut microbiota, changes in concentrations of metabolites produced by the gut microbiota, and diminished hepatic detoxification processes [49]. The authors noted that similar impacts on the gut microbiota in humans had been associated with increased cardiometabolic risk and stated that “NNS consumption during pregnancy and lactation may adversely affect infant metabolism” [49].

Adverse impacts on the maternal gut microbiota similar to those seen in animal studies would thus result in increased toxicant load systemically, as noted earlier [2], with increased toxicant exposures and oxidative stress for both the mother and the fetus. Correspondingly, a pro-oxidant environment could contribute to the dysbiosis of the gut microbiota. In the context of decreased GSH availability and increased vulnerability to oxidative stress and toxins, the overall result would be diminished neuroprotection. Assertions by Ayoub-Charette et al. that aspartame and its metabolites either do not reach critical organs or reach them in insufficient concentrations to affect either the mother or the child are refuted by the results from these earlier studies.

In our paper, we reported earlier findings of the presence of NNS in 65% of human milk samples [8], 77% of amniotic fluid samples [7], and 100% of cord blood samples studied [7], in order to highlight the extent to which these products are being consumed by pregnant women and to underscore their pervasive presence in the fluids to which both unborn children and infants are exposed. Even though intact aspartame cannot be measured in these fluids, the detection of the other two leading NNSs used in diet sodas and other diet beverages in all infant cord blood samples tested and in the large majority of amniotic fluid and milk samples underscores the striking degree to which offspring are now being exposed to these products during periods of profound neurodevelopmental vulnerability. Although aspartame does not persist intact in the body long enough to be detected in maternal blood or in these fluids, animal studies have repeatedly demonstrated that, through its metabolites, aspartame has pervasive influences on the health of both the consuming individual and offspring exposed in early life: either gestationally, through nursing, or both.

Our study is not the first to find associations between gestational/early-life exposures to aspartame or diet beverages and subsequent health risks in human offspring. Instead, our results follow and are congruent with those from an earlier case–control study [50], which found that maternal dietary methanol intake during pregnancy, calculated mainly from the consumption of aspartame-sweetened beverages and other products, was more than twice as high among the mothers of children with autism compared with the mothers of neurotypically developing children [50]. Our findings are also consistent with reports from large-scale prospective studies in finding significantly increased developmental risks among human offspring exposed in utero to diet sodas and other diet beverages [2]. These earlier cohort studies employed a prospective collection of dietary recall data and included covariates for several maternal cardiometabolic and dietary risk factors in their analyses. Among offspring gestationally exposed daily to diet sodas or to diet beverages in general, these earlier studies showed a 67% increased risk of early preterm delivery [51], a 57% increase in the risk of infants being large for gestational age at birth [52], an approximate doubling of the risk of being overweight or obese at the age of one year [53] and at the age of seven years [52], and a 30% increased risk of being diagnosed with asthma [54]. We hope that results from future analyses of data from large-scale prospective studies, with adjustments for additional risk factors, will soon become available to shed further light on the question of whether the daily maternal intake of diet sodas and/or aspartame, specifically, is associated with increased autism risk in exposed offspring. In the meantime, we would reiterate that while our results do not prove causation, they also do not stand alone: they are the latest in a long series of findings from both animal and human studies, suggesting that caution may be advisable for women considering the use of these products during pregnancy and breastfeeding. 

This study is also not the first to sound a warning about the potentially increased risk of developmental problems in offspring exposed to diet sodas, other NNS, and/or aspartame specifically. In 2018, the following warning appeared in a Science Advisory from the American Heart Association, entitled ‘Low-Calorie Sweetened Beverages and Cardiometabolic Health [55]: “On the basis of the available evidence, the writing group concluded that, at this time, it is prudent to advise against prolonged consumption of LCS [low-calorie-sweetened] beverages by children” [55]. In 2021, Laforest-Lapointe et al. wrote, “Our study suggests that infants exposed to ASB [artificially sweetened beverages] through their mothers may be at higher risk of shifts in microbial community structure related to early-life predisposition to metabolic diseases” [42]. In 2020, Palatnik et al. wrote, “Emerging evidence from animal studies warns against NNS consumption [in pregnant women] and present specific effects that harm the metabolism in offspring…As evidence accumulates and with about 30% of pregnant women self-reporting NNS consumption and possibly more with unintentional exposure, it is critical to question the safety of NNS consumption during pregnancy” [56].

Given the combined results from studies in both animals and humans in finding an increased risk of adverse health outcomes among offspring exposed gestationally to diet beverages, NNS, and/or aspartame specifically, we have chosen to follow the precautionary principle and add our voices to those of earlier investigators in encouraging pregnant women to carefully weigh the possible risks to their unborn children when considering the use of these products.
